# Relationship between rabbit anti-thymocyte globulin and development of PTLD and its aggressive form in renal transplant population

**DOI:** 10.1080/0886022X.2020.1759636

**Published:** 2020-05-19

**Authors:** Hatem Ali, Karim Soliman, Ahmed Daoud, Ingi Elsayed, Tibor Fülöp, Ajay Sharma, Ahmed Halawa

**Affiliations:** a Institute of Medical Sciences, Faculty of Medicine, University of Liverpool, Liverpool, UK; b Department of Medicine, Division of Nephrology, Medical University of South Carolina, Charleston, SC, USA; c Department of Renal Medicine, Methodist Hospital, Houston, TX, USA; d Department of Renal Medicine, Royal Stoke University Hospitals, Stoke-on-Trent, UK; e Medical Services, Ralph H. Johnson VA Medical Center, Charleston, SC, USA

**Keywords:** Induction therapy, complications, outcome, malignancy

## Abstract

**Introduction:**

The aim of our study is to explore the relationship of rabbit anti-thymocyte globulin (R-ATG) on development of post-transplant lymphoproliferative disease (PTLD) and its aggressive forms (monomorphic PTLD and Hodgkin lymphoma) in renal transplant recipients.

**Methodology:**

All patients diagnosed with PTLD post-renal transplant in the United States’ Organ Procurement and Transplantation Network from 2003 till 2013 and followed up till 2017 were retrospectively reviewed. Multi-variable logistic regression analysis assessed association of R-ATG to development of PTLD and its aggressive form.

**Results:**

Risk of developing PTLD post renal transplant is 1.35%. In comparison to interleukin-2 blocker induction therapy, R-ATG is associated with increased risk of development of PTLD (Odds Ratio = 1.48, confidence interval ranges from 1.04 to 2.11, *p* = .02) and is associated with higher risk of development of aggressive PTLD (Odds Ratio = 1.83, confidence interval ranges from 1.001 to 3.34, *p* = .04).

**Conclusion:**

We conclude that R-ATG induction is associated with a higher risk of PTLD and its aggressive form (monomorphic PTLD and Hodgkin lymphoma). Careful monitoring for development of PTLD in renal transplant recipients receiving R-ATG induction therapy is advised.

## Introduction

Post-transplant lymphoproliferative disease (PTLD) is one of the most common cancers occurring after solid organ transplantation, accounting for almost 20% of all cancers [1,2]. It is affected by the type and dose of immunosuppression regimens and is often associated with poor prognosis [3]. The average survival rates ranges between 25–30% [4]. Mortality rates can reach up to 80% among patients with monomorphic PTLD [5]. Those with T-cell lymphoma have the worst prognosis [6]. Induction immunosuppressive therapies, particularly rabbit anti-thymocyte globulin (R-ATG) has been reported to increase the risk of PTLD in solid organ transplant recipients and hematopoietic stem cell transplant (HSCT) [7–9]. However, a clear link of R-ATG to different types of PTLD remain scarce in the literature. PTLD can present as localized or disseminated disease [10]. The diagnosis is made by imaging and histopathology demonstrating lymphoproliferation [6]. In most of the patients, PTLD is induced by Epstein-Barr virus (EBV) infection [11]. Evidence of EBV DNA, RNA or protein may be demonstrated in tissues in case of EBV-positive PTLD [12]. According to the 2008 World Health Organization (WHO) classification there are 4 subtypes of PTLD: 1. Early hyperplastic lesions characterized by polyclonal B cell proliferation without malignant transformation presenting as infectious mononucleosis-like acute illness. 2. Polymorphic lesions with monoclonal or polyclonal lymphoid infiltrates, showing evidence of malignant transformation, but not fulfilling the criteria of any of the known lymphomas that occur in immunocompetent patients. 3. Monomorphic lesions with monoclonal lymphoid proliferations that fulfill the criteria for one of the lymphomas recognized in immunocompetent patients. Majority of monomorphic PTLD cases are Non-Hodgkin lymphoma of B-cell origin. Lastly, the fourth type named transplant-associated Hodgkin lymphomas, characterized by classic Reed-Sternberg cells in their lymph nodes [13]. Monomorphic PTLD and Hodgkin lymphoma are the most severe and aggressive forms of PTLD that require chemotherapy, radiotherapy and surgical intervention [5,6,14–17].

The initial treatment of PTLD is the reduction or withdrawal of immunosuppression which may lead to resolution of early lesions [18–20]. Other treatment options include immunoglobulin therapy, monoclonal antibodies, chemotherapy, radiotherapy and surgical excision [14]. R-ATG enhances the risk of PTLD *via* disrupting cancer immune-surveillance and immunological control of oncogenic viruses [21,22]. However, a clear link of R-ATG to different types of PTLD, particularly aggressive forms of PTLD (monomorphic PTLD and Hodgkin lymphoma) remains scarce in the literature. The aim of our study is to explore the relationship between R-ATG and development of PTLD and its aggressive forms (monomorphic PTLD and Hodgkin lymphoma) in renal transplant recipients. To our knowledge, this is the first and largest study to utilize UNOS database aiming to explore the association of R-ATG to monomorphic PTLD and Hodgkin lymphoma.

## Methodology

The study was exempt from ethical approval by Liverpool university. All renal transplant patients registered in organ procurement and transplantation network (OPTN) from January 2003 until January 2013, received R-ATG or interleukin-2 receptor antagonist induction therapies and discharged on calcineurin inhibitors maintenance therapy were retrospectively reviewed. Patients were followed up till June 2017. Exclusion criteria were patients who had transplants other than the kidneys, multiple organ transplant, previous renal transplant, patients who received treatment for post-operative acute rejection episodes, patients who received monoclonal antibody alemtuzumab (Campath) or any type of ATG for treatment of acute rejection till the end of follow-up period. Muromonab-CD3 antibody (OKT3) and high doses of R-ATG induction therapies have been commonly used in the USA in 1980s and early 1990s. However, afterwards, OKT3 has been commercially unavailable and lower doses of R-ATG has been widely used [23–28]. By 2003, the use of OKT3 has become very minimal [23]. Therefore, we excluded patients who had transplant before 2003. Also, patients who received T cell depleting agents other than R-ATG, historical induction therapies (alemtuzumab, OKT3, OKT4, eon, cyclophosphamide, mirozibine, nratg, brequinarsodium, xomazymecd5, anti LFA1, ICAM1, dab486il2, interleukin 1 antagonist, t10b9, interleukin 6 antagonist, anti TNF blockers, deoxyspergualin, everolimus, fty720, sirolimus), rituximab or mTOR inhibitors at time of discharge were excluded. Patients with missing date of transplant were excluded. Patients who had missing data about induction therapy or received dual induction therapy were excluded from analysis.

Collected data included recipient and donor age, sex, ethnicity, donor type (living/non living), extended criteria donors, type of induction therapy, maintenance immunosuppression medications at time of discharge, type of PTLD, panel reactive antibody (PRA) titer, cold ischemia time, HLA mismatches, EBV status, CMV, HBV, HCV and HIV status for the transplant recipient. Types of PTLD were defined according to the 2008 WHO classification [8]. Due to the aggressive characteristics of monomorphic PTLD and Hodgkin lymphoma, both types have been combined into one group for classification and analytical purposes in our study. As R-ATG and interleukin-2 receptor antagonist (IL-2RA) induction therapies are the 2 most common used agents for induction therapies, R-ATG was compared to IL2-RA while assessing its effect on development of monomorphic PTLD and Hodgkin lymphoma.

### Statistical analysis

STATA package-15 was used for the analysis. The starfiles used from OPTN database were kidpan, immunosuppression_discharge, immunosuppression_follow and kidney_malig_followup_data. Duplicates from each file were removed separately and then the files were merged into one file using 1:m merge command. Continuous variables were reported as means and standard deviation while categorical variables were reported as percentages or frequencies. To prove association, multi-variable logistic regression analysis was used to assess the impact of R-ATG induction therapy on the development of PTLD and aggressive PTLD (monomorphic PTLD and Hodgkin lymphoma) in the presence of other cohort characteristics that are well-known to affect development of PTLD. Performance and calibration of the model were assessed using AUC analysis and Pearson goodness-of-fit tests. *p* value less than .05 was a cutoff point for poor fit model. Patients with missing data about induction therapy were excluded while performing multi-variable logistic regression analysis. R-ATG was compared to IL2-RA induction therapy in the logistic regression models.

## Results

**Figure 1.**
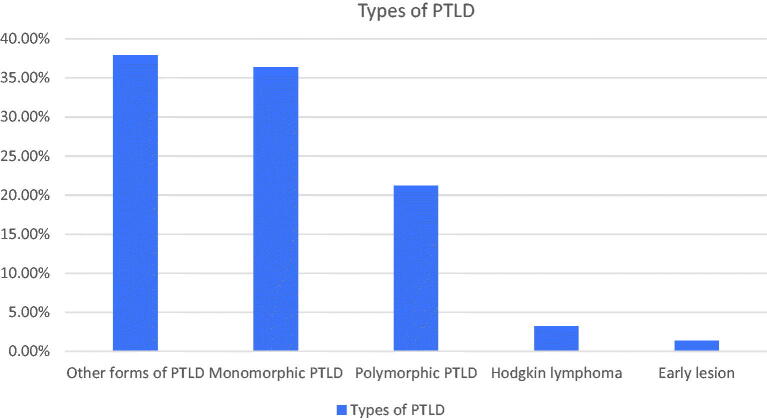
Types of PTLD.

After deduplicating selected files from OPTN database and removing patients who lack data about transplant date, 82838 patients were found to have renal transplantation since January 2000 till June 2017. Out of these patients, 1119 developed PTLD (1.35%). Types of PTLD are shown in Figure 1. Average time for diagnosing PTLD was 5.39 years post-transplant. Incidence of diagnosing PTLD one-year post transplant was 0.28%.

After applying inclusion and exclusion criteria to the merged files, sample size of the study was equal to 14,988 patients (8360 patients received R-ATG induction therapy and 6628 patients received IL2-RA induction therapy). Multi-variable logistic regression analysis assessing the impact of R-ATG induction therapy on development of PTLD is shown in Table 1. In comparison to IL2-RA induction therapy, R-ATG is associated with increased risk of development of PTLD (odds ratio = 1.48, confidence interval ranges from 1.04 to 2.11, *p* = .026). Area under the curve for this model equals 0.59. There is no evidence for poor fit in this model (*p* = 1).

**Table 1.** Relationship between R-ATG induction therapy development of PTLD.

	Odds ratio	*p* value	95% Confidence Interval
R-ATG versus IL2-RA induction	1.48	.02	1.04 to 2.11
Tacrolimus versus cyclosporine	0.87	.53	0.57 to 1.33
Sex:			
Male	1.08	.64	0.77 to 1.51
CMV:			
Positive	0.88	.48	0.62 to 1.25
EBV:			
Positive	0.69	.02	0.49 to 0.95
Recipient age	0.98	.08	0.97 to 1.00
Donor age	0.99	.61	0.98 to 1.00
HLA mismatch	0.92	.11	0.84 to 1.01
Steroids maintenance:			
Yes	1.50	.14	0.86 to 2.64

R-ATG: Rabbit antithymocyte globulin; IL2-RA: interleukin-2 receptor antagonist; CMV: Cytomegalovirus; EBV: Epstein-Barr virus; HLA: Human leucocyte antigen.

Multi-variable logistic regression analysis assessing the impact of R-ATG induction therapy on development of aggressive PTLD (monomorphic PTLD and Hodgkin lymphoma) is shown in Table 2. In comparison to IL2-RA induction therapy, R-ATG is associated with increased risk of development of PTLD (odds ratio = 1.83, confidence interval ranges from 1.001 to 3.34, *p* = .048). Area under the curve for this model equals 0.63. There is no evidence for poor fit in this model (*p* = 1).

**Table 2.** Relationship between R-ATG induction therapy development of aggressive PTLD (monomorphic PTLD and Hodgkin lymphoma).

Aggressive PTLD	Odds ratio	*p* value	95% Confidence interval
R-ATG versus IL2-RA	1.83	.04	1.001 to 3.34
Tacrolimus versus cyclosporine	0.63	.17	0.32 to 1.22
Sex:			
Male	1.08	.78	0.61 to 1.89
CMV:			
Positive	1.14	.67	0.61 to 2.10
EBV:			
Positive	0.84	.55	0.49 to 1.46
Recipient age	1.00	.89	0.98 to 1.02
Donor age	0.98	.09	0.96 to 1.00
HLA mismatch	0.88	.09	0.75 to 1.02
Steroids maintenance:			
Yes	0.79	.53	0.38 to 1.64

R-ATG: Rabbit antithymocyte globulin; IL2-RA: interleukin-2 receptor antagonist; CMV: Cytomegalovirus; EBV: Epstein-Barr virus; HLA: Human leucocyte antigen.

## Discussion

This retrospective study utilized the OPTN database and demonstrated that R-ATG induction therapy was associated significantly with increased risk of PTLD and a higher risk of developing the aggressive forms of PTLD. Our study analyzed a large cohort of PTLD-affected renal transplant recipients with 10-years recruitment period (2003-2013) allowing us to produce strong results. Similarly, previous studies utilized OPTN database to study the effect of immunosuppression (IS) on incidence of PTLD. Although concurring with our results, no study focused on subgroup analysis of different types of PTLD and its association with R-ATG use [29–31]. Also, we are one of the largest cohort study to assess the incidence of PTLD and its relation with induction therapy. Short recruitment period, with periods of less than 5 years is another valid limitation for the existing studies. In addition, previous registry data studies did not address important confounding factors like acute rejection. Furthermore, previous registry data studies were comparing ATG to no-induction or to old induction therapies like OKT3 and were including old types of ATG or not mentioning the type of ATG. We conquer in our study by using only R-ATG, excluding patients who received treatment for acute rejection with any type of ATG or with Campath. We also conquer by comparing R-ATG to IL2-RA as these are the most common induction therapies used nowadays. All these factors give more validity to our results. Dharnidharka *et al.*, showed that equine anti-thymocytic globulin (E-ATG) increased the risk of PTLD, while R-ATG did not [29]. This is possibly explained by the fact that E-ATG has been shown to be more potent and superior as compared to R-ATG and hence; with possible more risk of malignancies [32]. Although their finding is a point to consider, E-ATG is rarely used in renal transplant patients currently and our data reflect R-ATG effect which is more commonly used. Caillard et al., did a nation-wide study in France and included all newly diagnosed PTLD cases between 1998 and 2007 [33]. Concurring with our results, authors showed that T-cell based induction therapy was associated with higher risk of monomorphic PTLD. In addition, authors revealed that risk of developing brain lymphomas is four-fold higher in patients who received T‐cell depleting agents [24]. Interestingly, this may go in line with our findings, as diffuse B-cell lymphoma – commonest subtype of monomorphic lymphoma – is the commonest lymphoma affecting the brain. Due to insufficient data on tumor location, we were unable to examine this pattern on specific organs.

On the other hand, several other studies failed to show a link between ATG and PTLD. Cherikh *et al.*, found that polyclonal induction is not associated with a statistically significant higher risk of PTLD [34]. However, although they utilized the large OPTN database, again their short recruitment time of 3-years is a huge limitation to the latter study [34]. Moving forwards, Faull *et al.*, conducted a retrospective review on all PTLD patients after kidney transplant documented in the Australia & New Zealand Dialysis and Transplant Registry from 1970 to March 2003 [35]. They found that treatment with anti-T-lymphocyte antibodies per se was not associated with an increased risk of PTLD. However, in combination with calcineurin inhibitor (CNI), the use of anti-T-lymphocyte preparations was associated with an increased risk of PTLD. This could support our findings in some way, given that in the United States almost all immunosuppressive protocols are CNI-based. Additionally, these authors did not mention or analyze the correlation of IS regimen to different types of PTLD [35]. Quinlan *et al.*, used the UNOS registry to find the incidence & risk factors of early onset (within two years from date of transplant) & late onset PTLD (two or more years post-transplant) among kidney transplant recipients from 1999-2007 [36]. These authors found that T-cell induction was not associated with a higher risk of early or late onset PTLD. They also reported a greater proportion of late-onset monomorphic PTLDs compared to early-onset monomorphic PTLD, but they did not correlate different types of PTLD to induction therapies. In our study, we analyzed a recruitment period time of almost twice as long (15 vs 8 years), perhaps enabling us to capture the association between PTLD and its aggressive forms in relation to R-ATG therapy.

We used R-ATG at time of induction to ensure that it was administered at a specific time point before development of PTLD and its aggressive form (monomorphic PTLD and Hodgkin lymphoma). Furthermore, we excluded all patients that received treatment of post-operative acute rejection episodes or received Campath or any type of ATG for treatment of acute rejections during follow-up. This allowed us to exclude effect of treatment for acute rejection episodes as a confounding factor on our results. R-ATG can increase the risk of PTLD *via* disrupting cancer immunosurveillance and immunological control of oncogenic viruses and this concludes plausibility [21,22].

Our study is unique in several ways. We analyzed the association of R-ATG to different subtypes of PTLD and included a very large cohort of PTLD affected renal transplant recipients abstracted from the OPTN data. In addition, our long recruitment period of 10-years further enhanced our results. The present results are important in improving our understanding of immunosuppressive therapies and PTLD.

Limitations of using registry data include missing data and presence of non-measured confounders. Since monomorphic lymphoma is an aggressive disease mostly with poor outcome [37], we believe, none the less, that future use of depleting agents, including novel or more powerful depleting agents, has to be carefully and cautiously considered prior to administration.

## Conclusion

We conclude that R-ATG induction is associated with a higher risk of PTLD and its aggressive form (monomorphic PTLD and Hodgkin lymphoma). Careful monitoring for development of PTLD in renal transplant recipients receiving R-ATG induction therapy is advised.
